# A Novel Physeal-Sparing Fixation Technique for Tibial Eminence Fractures in Paediatric Patients

**DOI:** 10.7759/cureus.100191

**Published:** 2025-12-27

**Authors:** Ala Al-Qudah, Al-Muthana Al-Yamani, Mohammad Abushahot, Nizar Abu Alannaz, Mutaz Ghabashneh, Naser Shari

**Affiliations:** 1 Orthopedic Surgery Department, Sports and Arthroscopy Division, Jordanian Royal Medical Services, Amman, JOR

**Keywords:** knee instability, physeal-sparing fixation, proximal tibial physis, tibial eminence fractures, transphyseal drilling

## Abstract

Introduction

Tibial eminence fractures are common knee injuries in paediatric patients. They are an uncommon cause of knee injury that is associated with effusion, and most frequently present in the paediatric population. These injuries affect knee stability. Surgical management is challenging and associated with risks and complications due to the direct relation to the proximal tibial growth plate. We developed a new physeal-sparing technique to fix these fractures while avoiding any violation of the proximal tibial growth plate.

Materials and methods

This new technique involves transfixing the avulsed tibial eminence using multiple non-absorbable high-tension sutures passed through the anterior cruciate ligament (ACL) in a crossed fashion, then creating subcapsular tracts deep to the anterior capsule, passing the sutures through these tracts, and securing them to the anterior tibial surface distal to the growth plate using anchors. Post-operative adjustable knee brace was applied, and patients were allowed to fully weight bear with crutches, and a full range of motion was allowed. All patients were referred to rehabilitation for cryotherapy, quadriceps muscle strengthening, and passive and active range-of-motion exercises postoperatively for six weeks; thereafter, resisted closed-chain exercises were commenced for three months before return to sports. All patients were followed for two years using clinical assessment, serial radiographs, and Lysholm scores.

Results

Twelve patients who were acutely referred to our clinic underwent this new tibial eminence fixation technique between 2020 and 2023. All patients had type III or IV tibial eminence fractures according to the modified Meyers and McKeever classification. At presentation, all had swollen knees with limited range of motion and demonstrated positive Lachman and anterior drawer tests when examined under anaesthesia. Arthroscopy confirmed the diagnosis, and all fractures were fixed using No. 2 FiberWire sutures (Arthrex, Naples, FL, USA) in a crossed fashion, secured to the tibia distal to the physis with two 4.5-mm SwiveLock anchors (Arthrex). Reduction was performed before fixation, and satisfactory reduction was achieved in all cases. Postoperatively, all patients had intact neurovascular examinations and were able to start quadriceps strengthening and range-of-motion exercises in the first week. None developed early or late postoperative complications, and all showed signs of healing on radiographs by six weeks, allowing initiation of resisted exercises. All patients returned to their preinjury level by six months, and none showed clinical or radiographic evidence of growth-plate injury or arrest.

Conclusions

This novel physeal-sparing tibial eminence fixation technique has shown that it is a safe fixation method with no short-term complications and has achieved healing in all patients who had the procedure. Further long-term studies and clinical trials are required to compare the results with other fixation methods that are currently in use.

## Introduction

Tibial eminence fractures, or tibial spine fractures, represent avulsion injuries of the anterior cruciate ligament (ACL) insertion on the tibial plateau. These injuries are relatively common in the paediatric population due to ligaments' increased elasticity compared to the weaker developing bone [1]. They account for approximately 2-5% of knee injuries associated with joint effusion, with an estimated incidence of 3 per 100,000 children annually [2,3]. The peak incidence occurs between the ages of eight and 14 years, coinciding with periods of skeletal growth and increased participation in sports activities.

First described by Meyers and McKeever [1] in 1959, tibial eminence fractures have been classified into four types based on the degree of fragment displacement and comminution [4]. Type I fractures are nondisplaced, type II fractures involve anterior elevation with the posterior hinge intact, type III fractures are completely displaced, and type IV fractures exhibit comminution [4]. Treatment aims to restore normal ACL tension, re-establish knee stability, and prevent long-term sequelae such as instability, arthrofibrosis, or growth disturbances [5].

Management strategies depend on the fracture type and the patient's skeletal maturity. Nonoperative management may suffice for type I fractures, but displaced fractures (types II-IV) typically require surgical intervention [6]. Traditional fixation methods have included open reduction with internal fixation using cannulated screws, which provide strong mechanical stability but risk iatrogenic injury to the proximal tibial physis [7]. More recently, arthroscopic techniques employing suture fixation through transphyseal tunnels have gained popularity, although concerns about growth plate injury persist [8].

To overcome these challenges, physeal-sparing techniques have been developed to avoid transphyseal drilling while achieving secure fixation. Among these, the use of arthroscopic suture bridge constructs with knotless anchors represents a significant advancement [9]. However, issues related to reduction quality, fragment stability, and vector of pull during fixation still present technical hurdles.

In response to these challenges, we developed a novel physeal-sparing fixation technique that involves passing high-tension sutures through the ACL in a crossed configuration, routing them subcapsularly beneath the anterior capsule, and securing them distal to the physis using knotless anchors. This method aims to provide stable anatomical reduction while completely preserving the integrity of the proximal tibial growth plate. The present report describes the surgical technique and our early clinical experience with this approach. This study aims to describe the surgical technique and report early clinical outcomes of a novel arthroscopic physeal-sparing fixation method for tibial eminence fractures in pediatric patients.

## Materials and methods

This report outlines a novel surgical technique for the physeal-sparing fixation of tibial eminence fractures in pediatric patients. Developed at the Royal Medical Services by Dr. Almuthanna Alyamani and utilised in 12 cases since 2020, the technique prioritises anatomical reduction and stable fixation without compromising the proximal tibial growth plate. We included skeletally immature patients who sustained an acute isolated tibial eminence fracture that is amenable to arthroscopic fixation. We excluded patients who presented late (i.e., after three weeks), were associated with other injuries, or required an open procedure. The procedure involves fixation of the avulsed tibial eminence using multiple non-absorbable high-tension sutures passed through the ACL in a crossed configuration. These sutures are then routed through subcapsular tracts beneath the anterior capsule and secured to the anterior tibial surface distal to the growth plate using anchors.

Postoperatively, patients are allowed full weight-bearing with the assistance of crutches while wearing an adjustable knee brace, permitting a full range of motion. All patients are enrolled in a rehabilitation program including cryotherapy, quadriceps strengthening, and passive and active range of motion exercises for six weeks. This is followed by a three-month closed-chain resistance exercise regimen before resuming sports activities. Given the descriptive nature of the study and the small sample size, no formal statistical analysis was performed.

## Results

Our case series and clinical experience

Since 2020, our team has embarked on using a novel physeal-sparing fixation technique for paediatric patients presenting with tibial eminence fractures. Over this period, we treated 12 young patients, all boys, who sustained their injuries during various sports activities. The average age among the group was 12.8 years, ranging from as young as eight to as old as 15. None of these children had a history of previous knee injuries or any significant underlying medical conditions.

Typically, these patients arrived at our institution following urgent referrals from other hospitals. Initial stabilisation at the referring centres involved immobilising the knee in extension with a backslab. Upon arrival, each patient underwent a thorough assessment, including a thin-slice CT scan to accurately evaluate the fracture. According to the Modified Meyers and McKeever Classification, five of the cases were identified as type III or III+ fractures, while the remaining three were classified as type IV.

Before surgery, every child was examined under anaesthesia. The findings were remarkably consistent: each had a grade III knee effusion and positive results on the Lachman, anterior drawer, and pivot-shift tests - all indicating significant anterior instability. Despite this, stress testing for varus and valgus instability at 30 degrees of knee flexion showed stable ligaments, and all patients retained full passive range of motion.

During diagnostic arthroscopy, we confirmed that the fractures were isolated to the tibial eminence, with no associated soft tissue injuries. Interestingly, every case revealed cartilage comminution and fragmentation that had not been apparent on the initial CT scans. However, these findings did not necessitate a change in our surgical approach.

Using our physeal-sparing arthroscopic technique, we achieved anatomical reduction, restored the ACL tibial footprint, and secured stable fixation in all cases. Intraoperative testing at the conclusion of each procedure confirmed a full range of knee motion and restored stability, with negative results on the Lachman, anterior drawer, and pivot-shift tests.

Recovery was smooth for nearly all patients. Only one child developed postoperative knee stiffness due to non-compliance with physiotherapy and required manipulation under anaesthesia four weeks later. There were no cases of infection, readmission, or wound complications.

We followed each patient for a minimum of two years, or until they had safely returned to sports. None showed any clinical or radiographic evidence of growth disturbance or physeal injury. At their final follow-up, every patient had regained full knee stability and function, resuming sports within a year post-surgery.

Surgical technique: a stepwise approach

Our surgical technique is specifically intended for skeletally immature patients who have an open proximal tibial physis and a tibial eminence fracture. It is suitable for both simple and comminuted fractures, as the method relies on traction through the ACL rather than direct bony fixation.

Preoperative assessment

Most often, patients are referred urgently from other hospitals, having already undergone initial stabilisation. Upon admission, we conduct a comprehensive evaluation, including a detailed history, clinical examination, and advanced imaging. We carefully document the mechanism of injury, the patient’s activity level before injury, and any relevant medical or surgical history.

Physical examination typically finds the patient in a backslab cast. We make it a point to remove the cast and thoroughly assess the soft tissues. Neurovascular status is carefully documented, though assessing knee stability can be challenging in an awake, injured child.

A CT scan is routinely performed to accurately characterise the fracture and aid in surgical planning. MRI is reserved for situations where additional injuries or occult fractures are suspected. All patients and their guardians provide informed consent prior to arthroscopic or open fixation of the tibial eminence.

Patient positioning

In the operating room, the patient is placed supine, with a high-thigh tourniquet applied over padded stockings and a wool bandage. The foot is positioned flush with the end of the table, and a bolster is used to facilitate full flexion and extension of the knee. We position the knee at 90 degrees of flexion, with a slight valgus and external rotation of the hip. A lateral thigh support at mid-thigh assists in opening the medial compartment and allows for the figure-of-four position, providing easy access to the lateral compartment. An examination under anaesthesia is then performed to assess joint range of motion and stability.

Diagnostic arthroscopy

Once the patient was positioned and prepped, we began by marking the key surface landmarks around the knee. With these guides in place, we established a standard high anterolateral portal, carefully selected for its ability to provide a broad, panoramic view of the tibial eminence. This vantage point allowed us to work without the risk of our instruments crowding the joint space, ensuring clarity and manoeuvrability throughout the procedure.

As is often the case in these injuries, we encountered hemarthrosis, blood within the joint, that clouded our view. To address this, we created an anteromedial portal under direct visualisation, which facilitated the drainage of fluid and markedly improved the clarity of the arthroscopic field. In some situations, we found it helpful to add a central portal, particularly when additional assistance was needed for fracture reduction and fixation.

We proceeded with a meticulous arthroscopic exploration of the joint, taking care to fully delineate the nature of the fracture. It was essential not only to assess the fracture itself but also to look for any involvement of the anterior horn of the lateral meniscus, which can sometimes become entrapped within the avulsed bone fragment. We also remained vigilant for other significant intra-articular injuries that might alter our surgical plan.

Fracture identification and bed preparation

**Figure 1:**
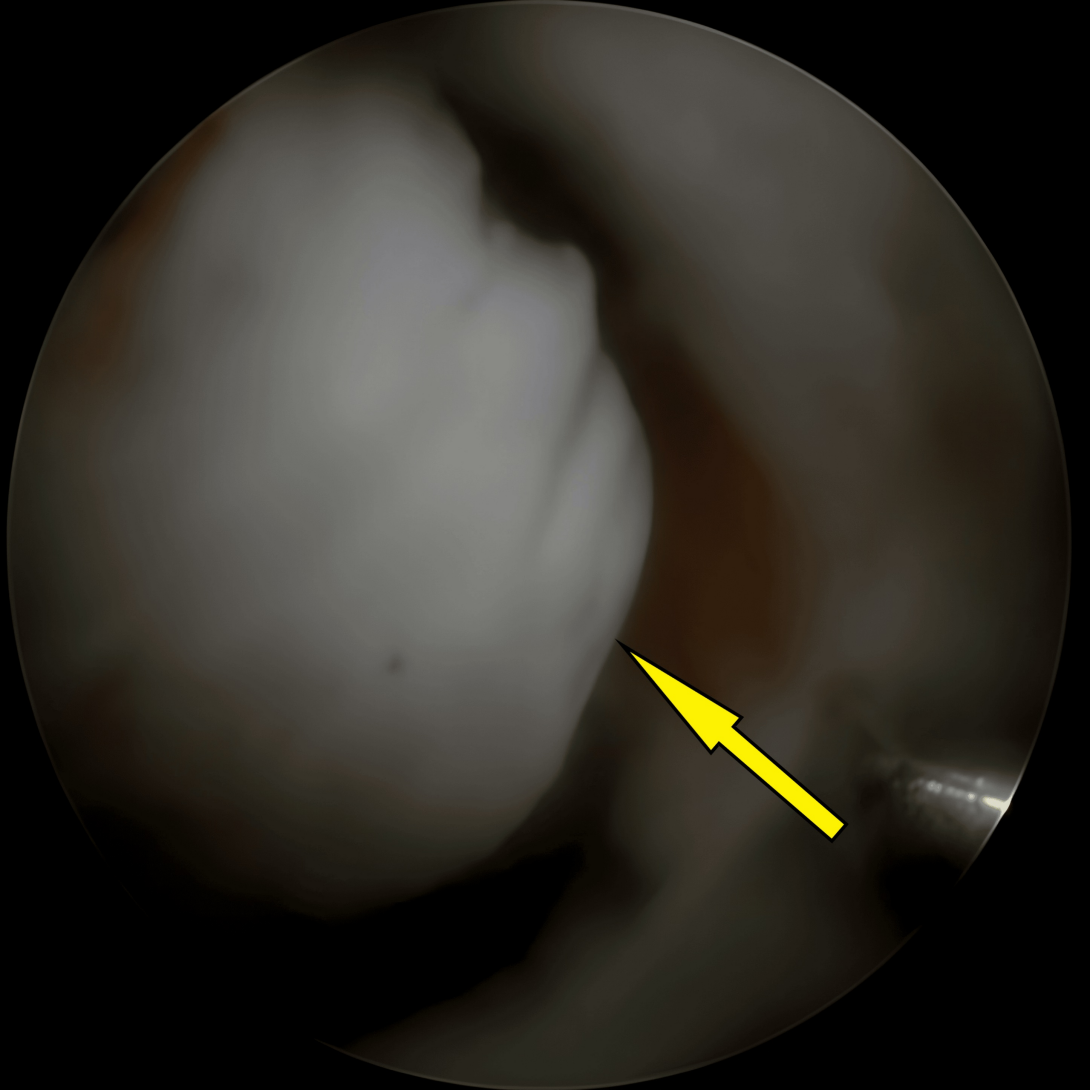
An avulsed tibial eminence encountered initially once knee scope started, before any preparation for avulsed fracture (arrow).

**Figure 2:**
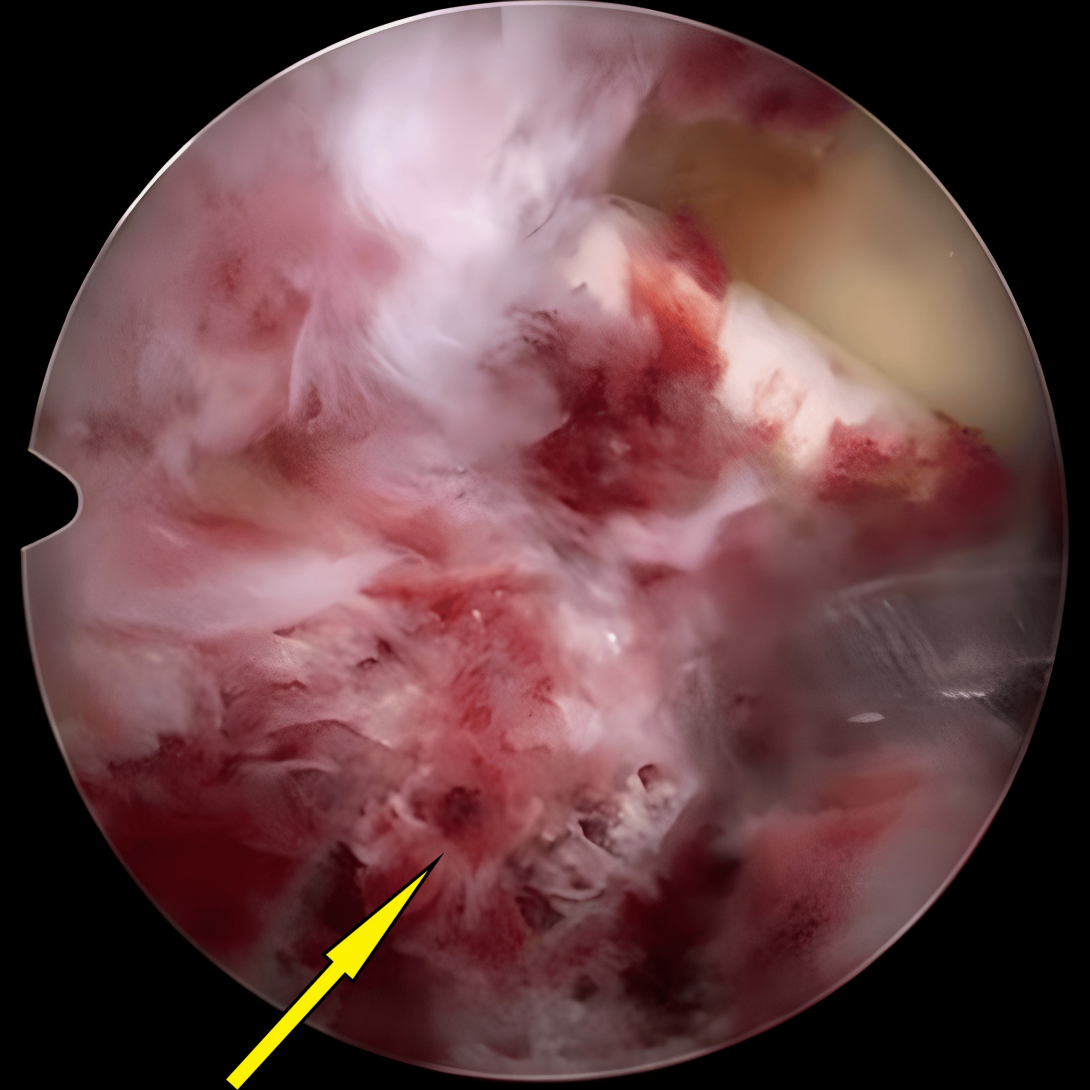
Avulsed tibial eminence bed before preparation and hematoma clearance (arrow).

**Figure 3:**
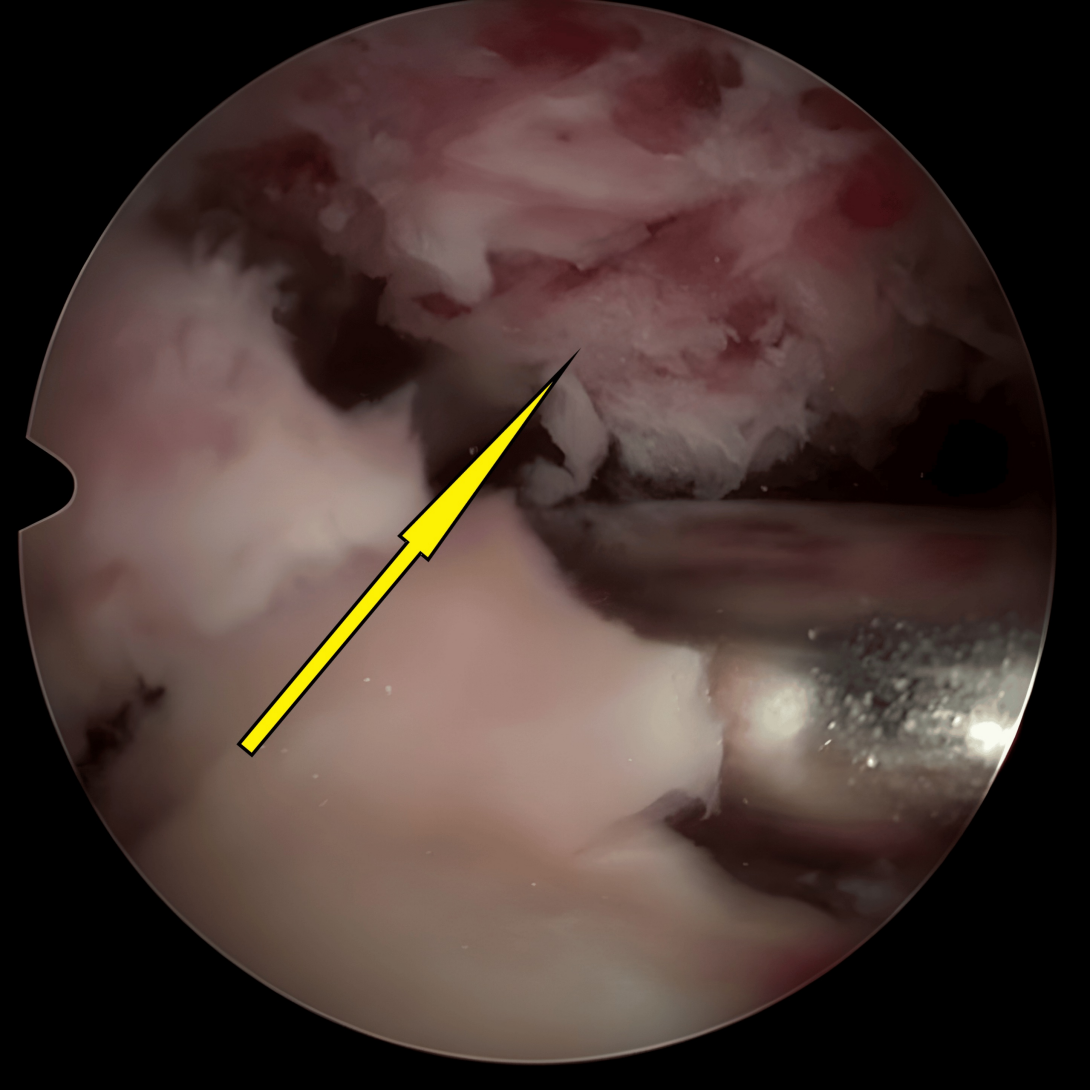
Initial stages of bed preparation for the avulsed tibial eminence, during which the hematoma was cleared (arrow).

With the joint fully visualised, our attention shifted to the fracture bed (Figure 1). We gently cleared away any hematoma and loose bone fragments that had accumulated in the area (Figures 2-3). Careful identification and preparation of the fracture edges were crucial steps in facilitating an accurate reduction. Particular attention was paid to the surrounding soft tissues; we worked methodically to clear the synovium and navigated around the intermeniscal ligament and anterior horn of the medial meniscus, always mindful to avoid causing any iatrogenic injury.

**Figure 4:**
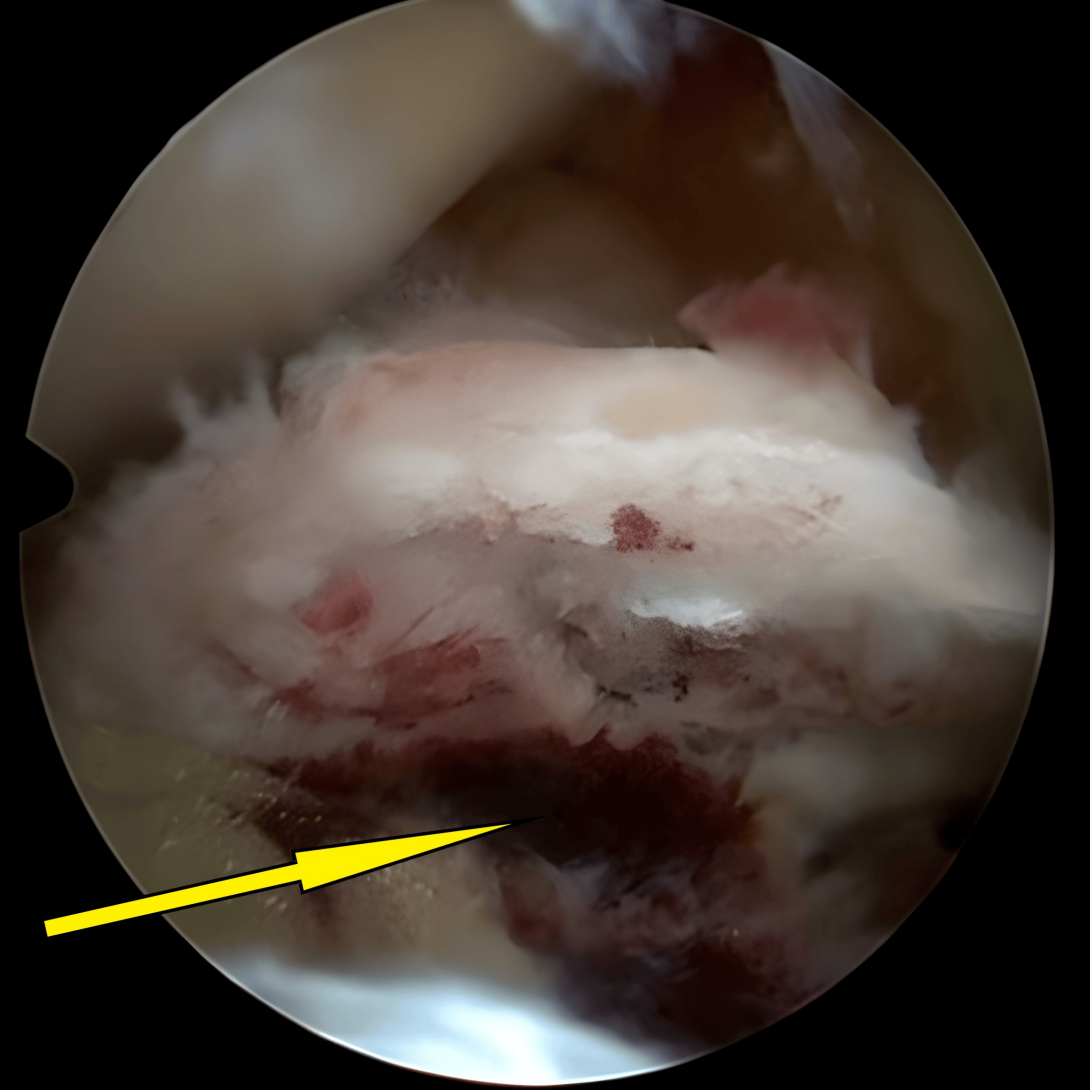
Avulsed tibial eminence bed after preparation and hematoma clearance, ready for reduction (arrow).

Occasionally, judicious debridement of the fat pad was necessary to achieve optimal visualisation. However, we were careful to limit this step, recognising that extensive debridement was rarely needed and could be detrimental. By taking these measures, we ensured clear visualisation of the anterior edge of the fracture (Figure 4), enabling us to control the reduction with greater precision, just as illustrated in Figures 1-4.

Fracture reduction and subcapsular tunnel preparation

The fracture reduction began with the use of a blunt trocar introduced through the central portal. As the surgeon guided the instrument into place, an assistant carefully maintained the newly achieved reduction, ensuring that the bone fragments remained aligned until the fixation was completed. In more complicated scenarios, such as when the fracture was comminuted, the bed was unprepared, or the injury was old and complicated by adhesions to the ACL, the surgical team first had to perform a thorough synovial release and remove any obstacles that could hinder the reduction.

**Figure 5:**
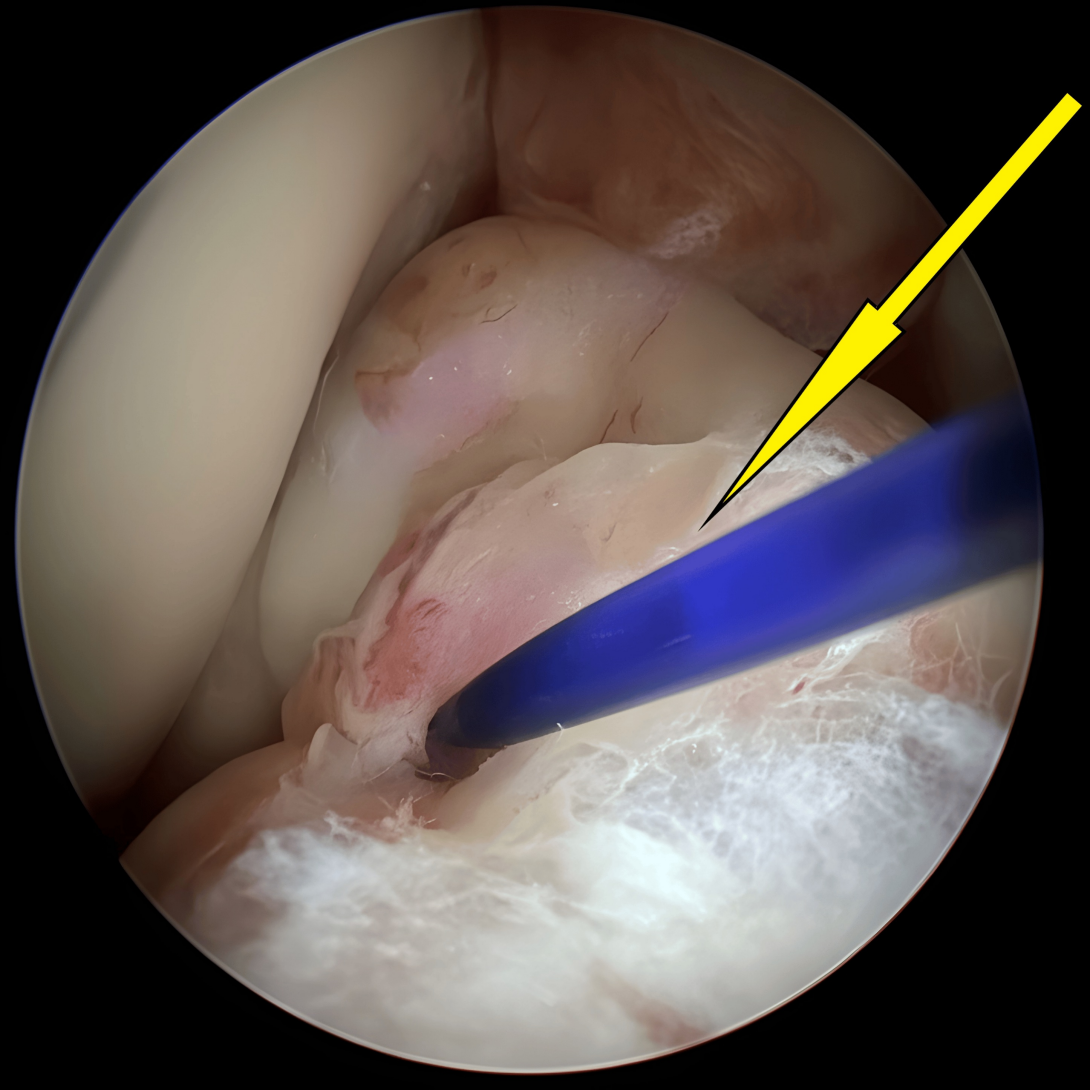
A polydioxanone (PDS) suture looped around the anterior cruciate ligament (ACL) using a lasso technique to aid fragment reduction before exchanging it for FiberWire (arrow).

**Figure 6:**
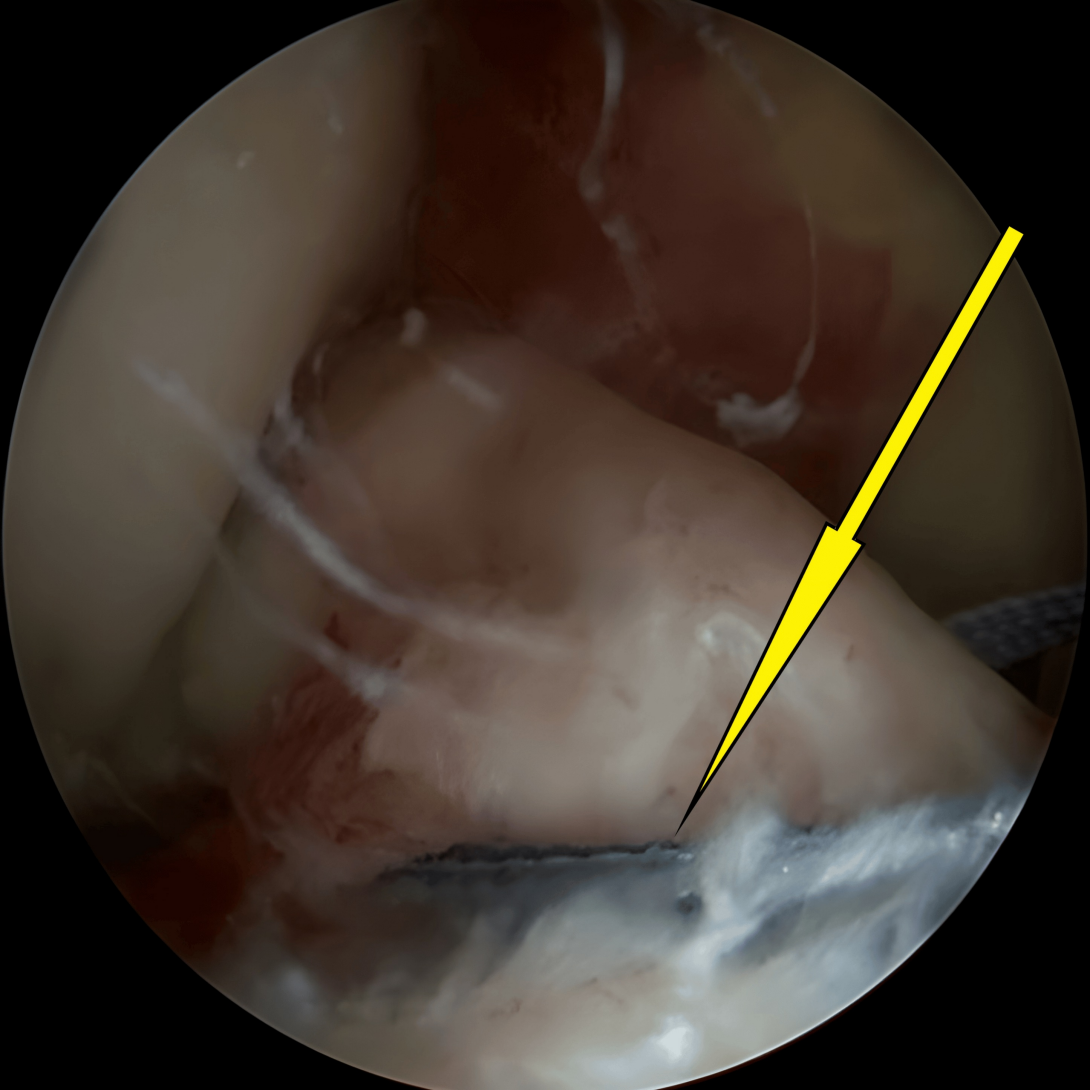
A high-tensile No. 2 FiberWire suture looped around the anterior cruciate ligament using a lasso technique in a luggage-tag configuration to aid fragment reduction (arrow).

With the area prepared, the surgeon selected a high-tensile suture, typically No. 2 Ethibond (Ethicon, Somerville, NJ, USA) or FiberWire (Arthrex, Naples, FL, USA), and looped it around the base of the ACL just above the avulsed bone fragment. This was performed using a lasso technique in a simple configuration to draw the fragment back into position (Figure 5). Correct suture positioning was crucial, as it allowed controlled traction on the fragment without risking damage to the ACL (Figure 6).

**Figure 7:**
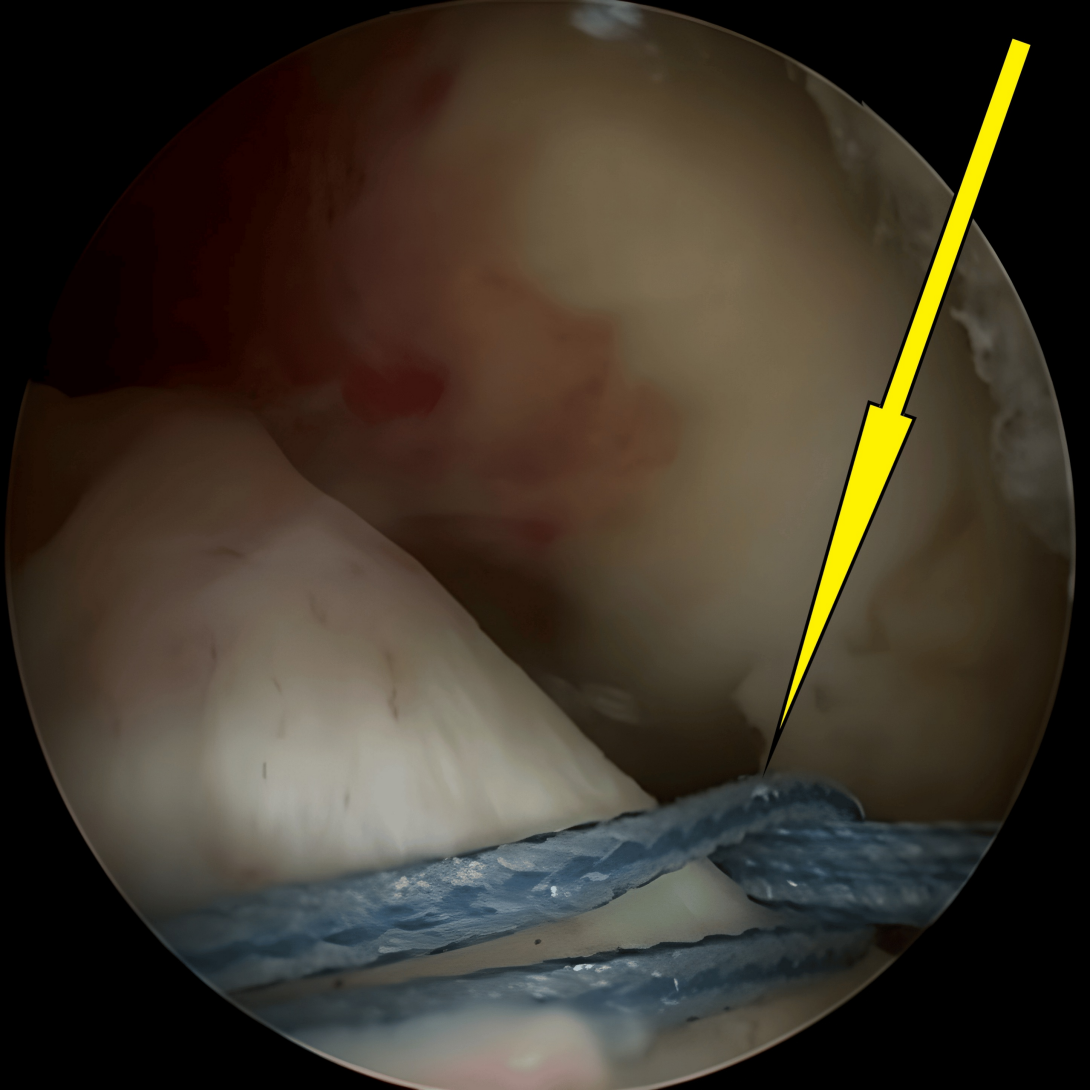
Luggage-tag configuration of the FiberWire used for the first suture passed around the anterior cruciate ligament to aid fragment reduction (arrow).

**Figure 8:**
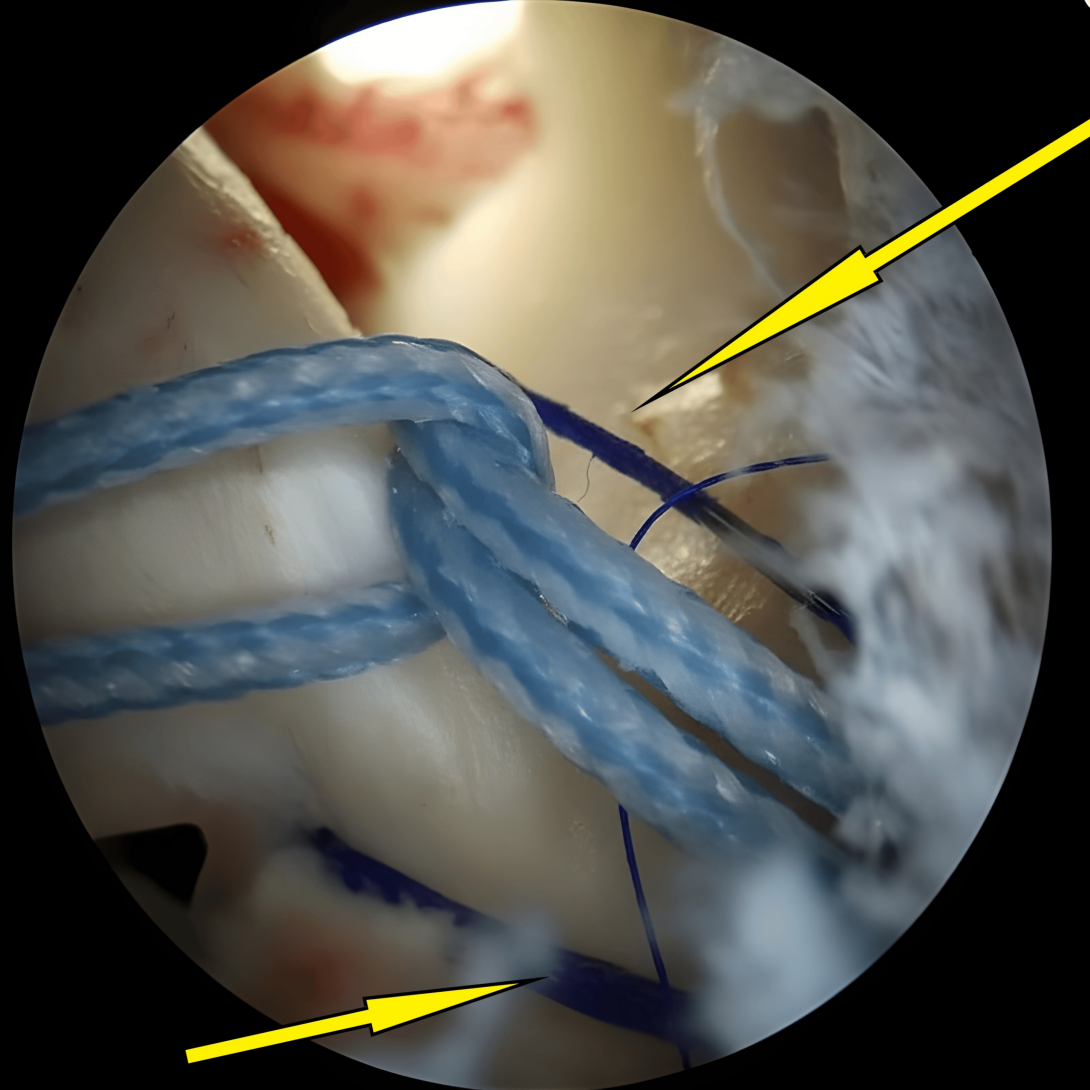
A polydioxanone (PDS) suture looped around the anterior cruciate ligament using a lasso technique to aid fragment reduction before exchanging it for the second FiberWire (arrows).

**Figure 9:**
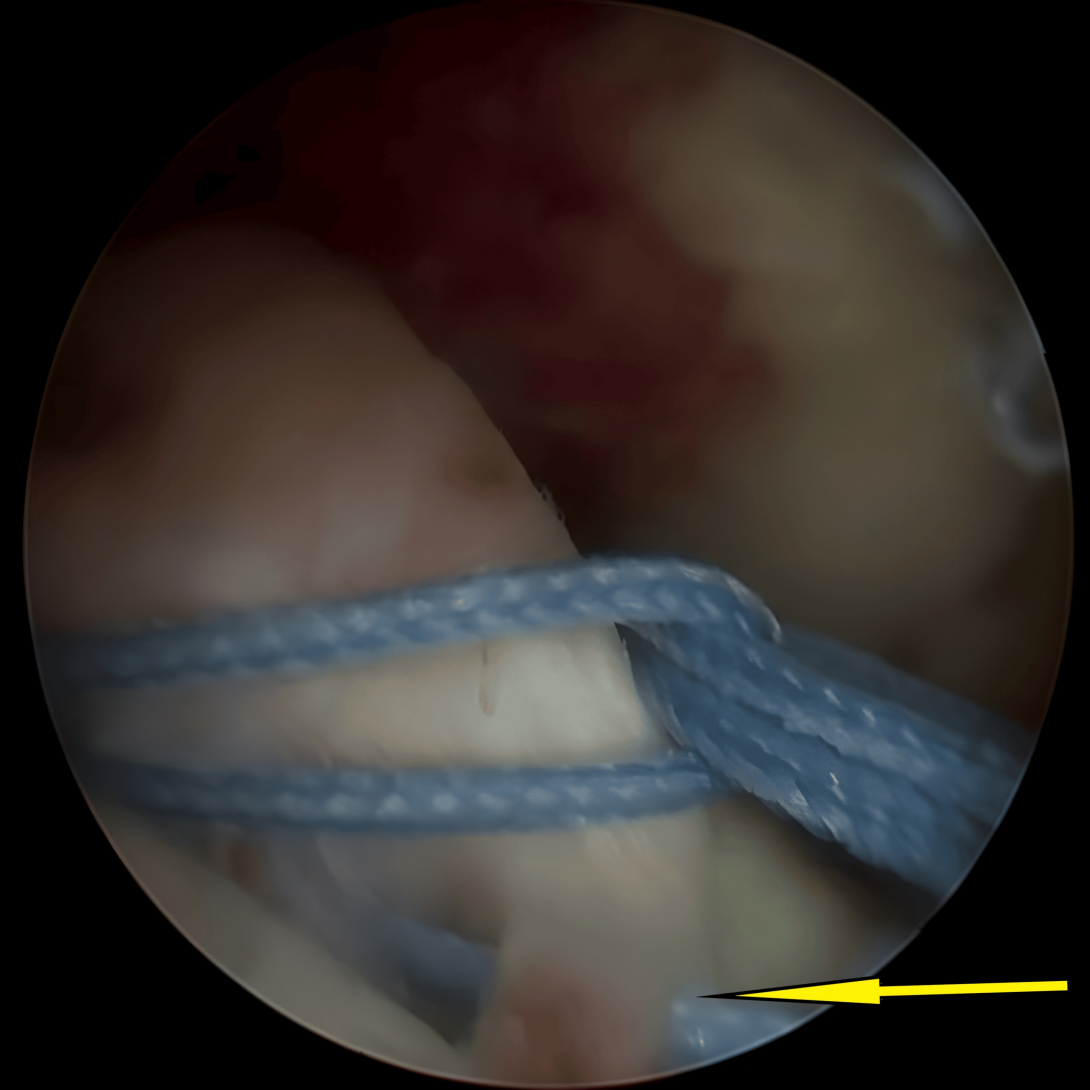
A second high-tensile No. 2 FiberWire suture looped around the anterior cruciate ligament using a lasso technique in a luggage-tag configuration to aid fragment reduction (arrow).

**Figure 10:**
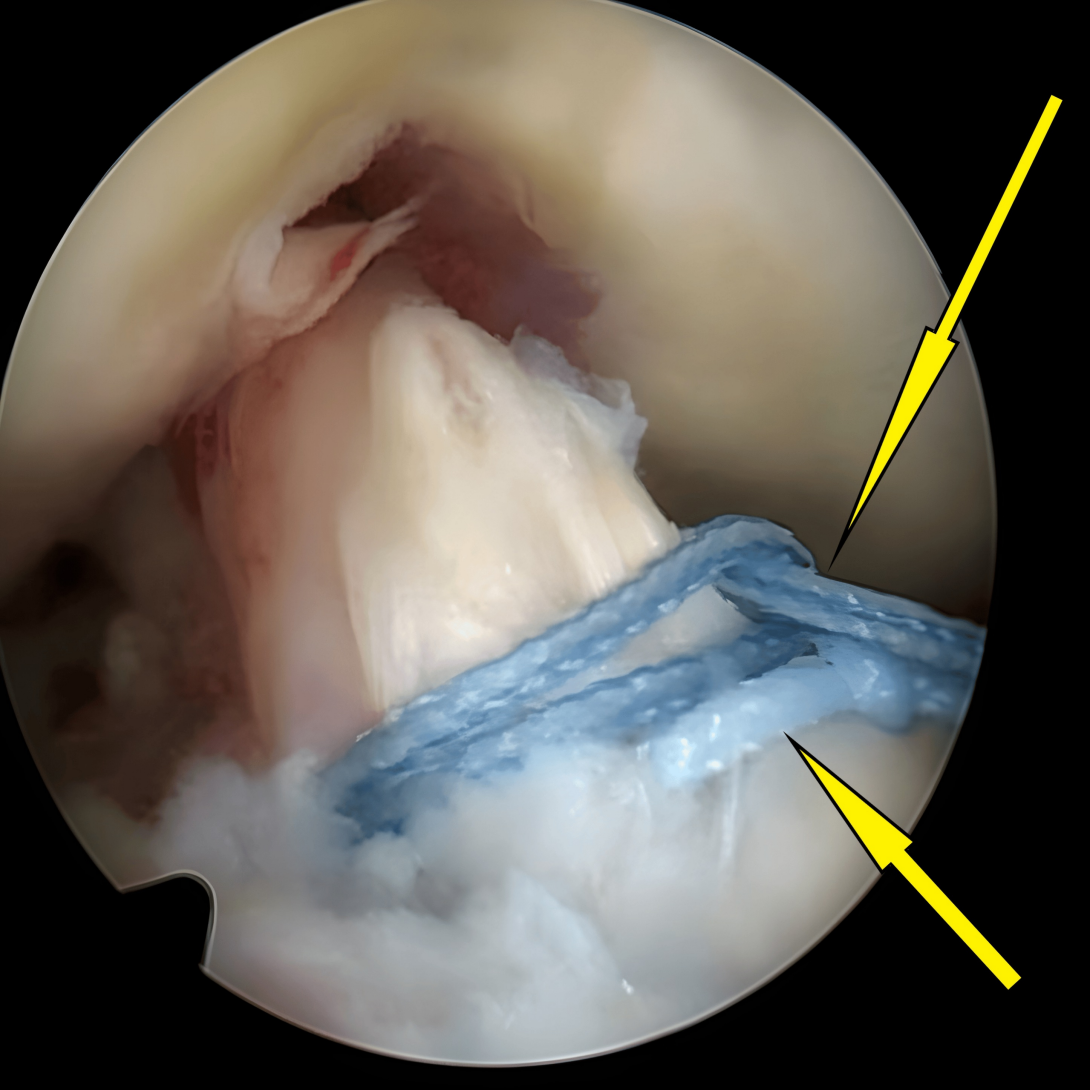
Final configuration of two No. 2 FiberWire sutures looped around the anterior cruciate ligament before fixation with a knotless anchor (arrows).

In most cases, two sutures were placed, ensuring a firm grasp on the fragment and providing the means for definitive fixation later in the procedure (Figures 7-8). Throughout this process, preserving the integrity of the ACL was paramount. The surgeon paid close attention to the size of the lassos and the distance between each suture, making sure that the sutures would not cut through the tissue during reduction (Figures 9-10). This careful, methodical approach ensured both the success of the reduction and the long-term stability of the repair (Figures 5-10).

**Figure 11:**
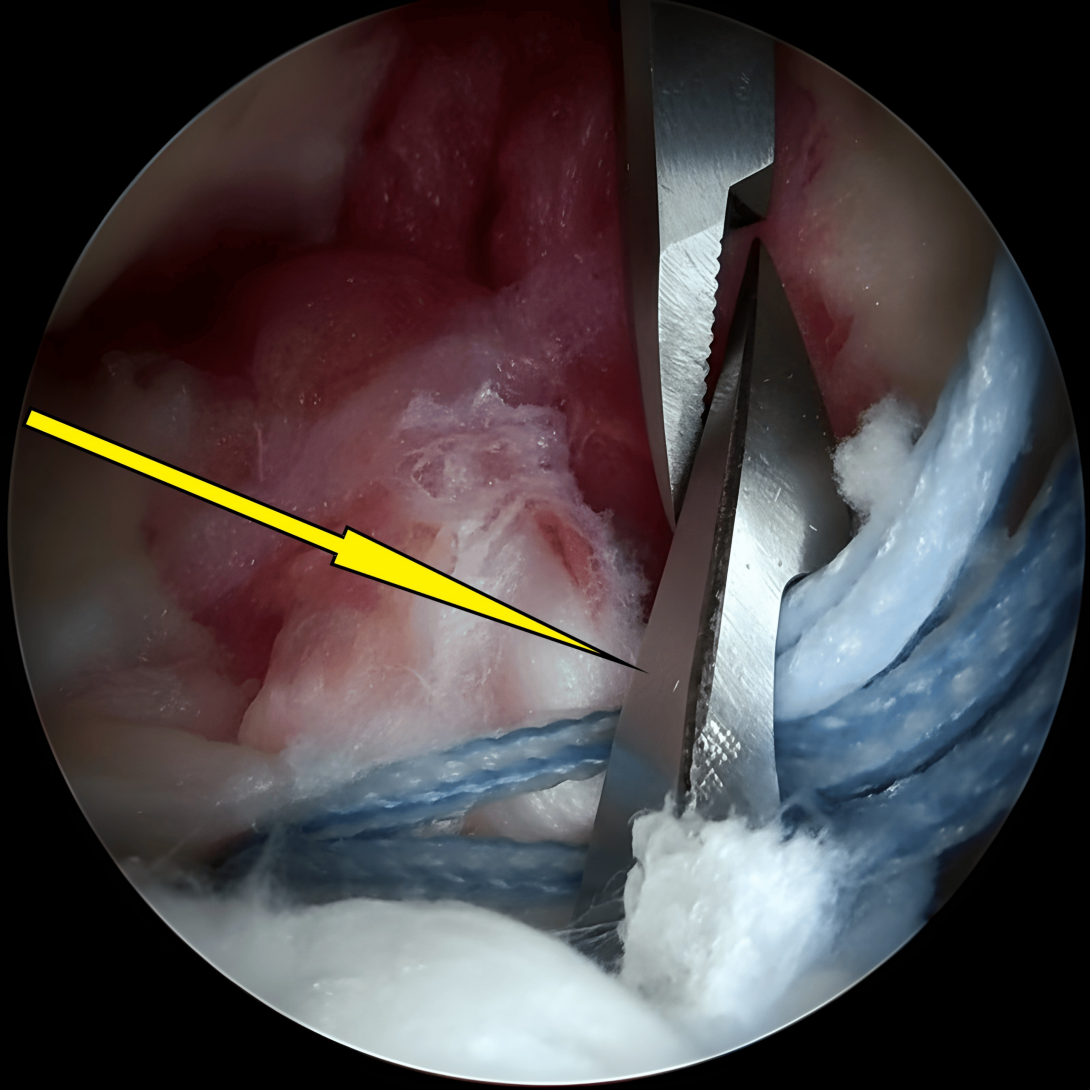
Passage of the suture retriever from distal to proximal through the subperiosteal, subcapsular tunnel, allowing the previously placed sutures to be shuttled and pulled distally (arrow).

Once the fracture reduction was secure, the focus shifted to creating a subcapsular tunnel for anchor placement (Figure 11). The procedure began with a careful 2-cm transverse incision, made just above the pes anserinus insertion and centred on the subcutaneous anteromedial surface of the tibia. This incision provided the necessary working space for future anchor placement, all while minimising the risk of injury to the medial collateral ligament (MCL) or the growth plate (physis).

With the initial incision completed, the surgeon applied superior traction to the tissues and made a second, smaller incision - about one cm in length - at the most distal part of the knee capsule, aiming towards the centre of the knee. In this approach, it wasn’t necessary to peel the capsule away from the bone; instead, the incision was made as close as possible to the capsule’s attachment, just large enough to permit the passage of a probe or suture retriever.

Next, the suture retriever was advanced from the distal to the proximal end through the newly created tunnel. This allowed the surgeon to shuttle the previously positioned sutures through the tunnel, pulling them distally. With a gentle downward pull on the sutures, the avulsed bone fragment was guided back towards its anatomical bed, while a trocar inserted through the medial working portal helped maintain the reduction.

Throughout this process, constant pressure was applied to the fragment, ensuring that it remained in position until the final fixation was secured. By passing the sutures anterior to the ACL footprint, the surgeon was able to provide compression on the front portion of the avulsed fragment, which effectively prevented any anterior displacement or the risk of the intermeniscal ligament becoming trapped (Figure 11).

Fracture fixation

After achieving a satisfactory reduction, the next step was to secure the sutures to the anterior tibial metaphysis. This was accomplished using two far-spread, knotless anchors, either 3.5 mm or 4.5 mm in size. The placement of these anchors demanded precision, and fluoroscopic guidance was used throughout to ensure that the growth plate (physis) remained unharmed.

**Figure 12:**
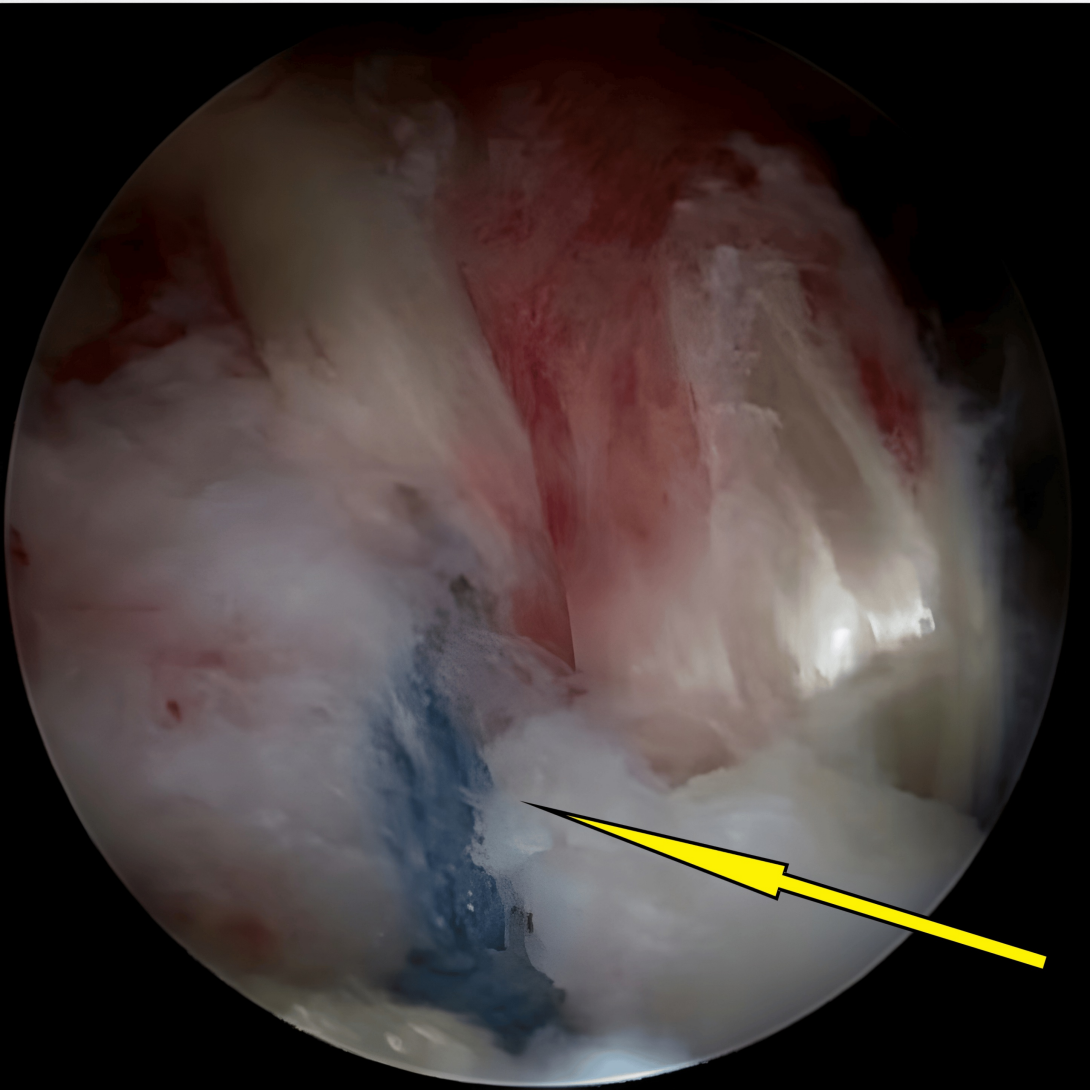
Sutures secured to the anterior tibial metaphysis using a single 3.5-mm knotless anchor (arrow).

**Figure 13:**
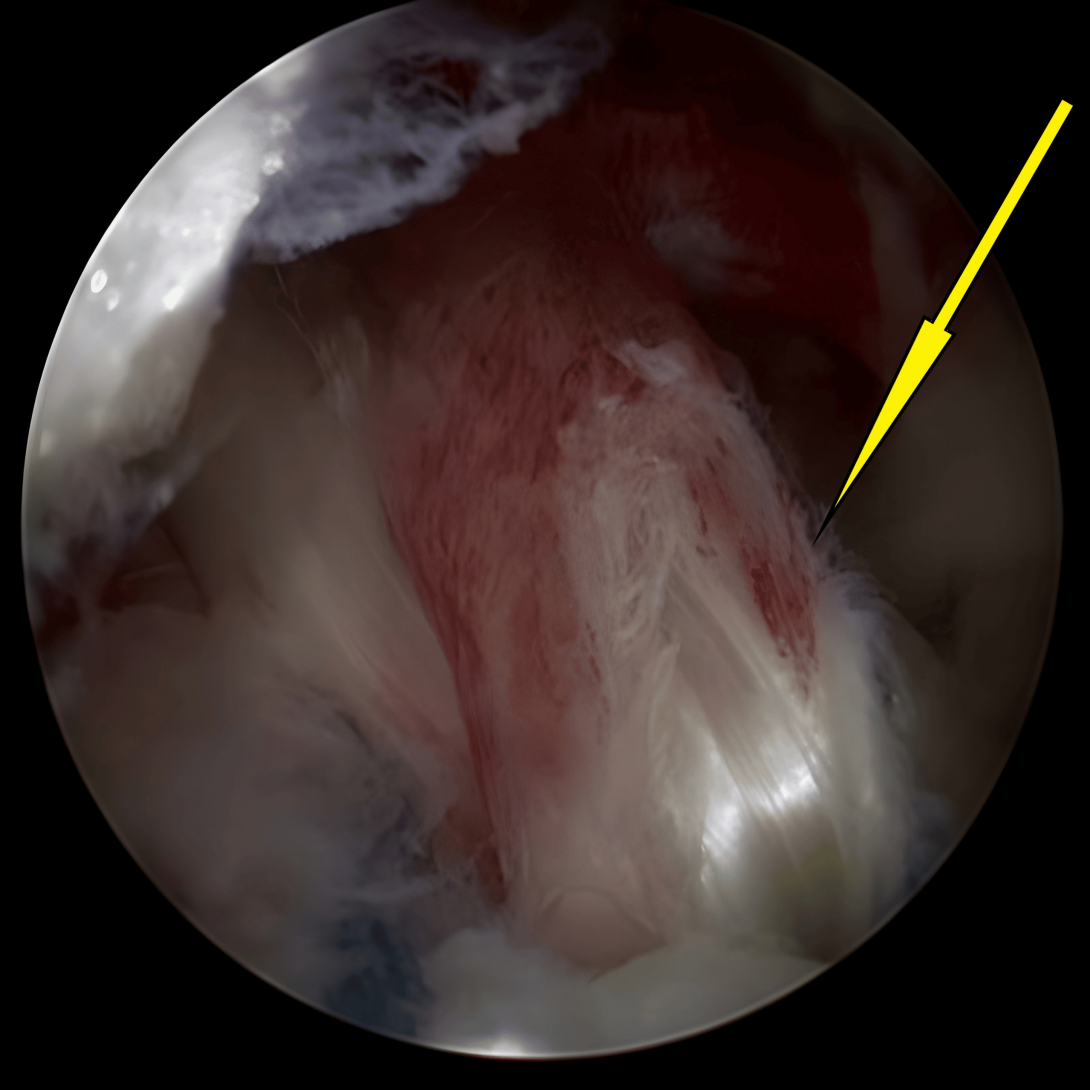
Final fixation of the anterior cruciate ligament after the sutures are secured to the anterior tibial metaphysis using a single 3.5-mm knotless anchor (arrow).

Once the anchors were in place, the surgical team reassessed the stability of the fracture and the tension of the ACL. This was done both arthroscopically (Figures 12-13) and clinically, using a probe to check the repair. To further confirm the integrity of the reconstruction, clinical tests, including the Lachman, anterior drawer, and pivot-shift tests, were performed.

If any instability was detected during these assessments, it was promptly addressed by placing additional sutures as needed, following the same careful technique described earlier (Figures 12-13).

Wound closure and dressing

With the fixation stable and secure, the surgical team turned their attention to closing the wounds. Using 3-0 Monocryl sutures (Ethicon, Somerville, NJ, USA), they carefully approximated the skin, ensuring a neat closure. The knee was then gently dressed with layers of gauze, wool, and crepe bandages to protect the surgical site. To further safeguard the repair, a backslap cast was applied, holding the knee in full extension for the first two weeks.

Postoperative protocol

Following surgery, patients typically remained in the hospital for 24 hours. During this time, a postoperative CT scan was performed to confirm that the reduction was satisfactory. Once the patient was fully awake and cooperative, physiotherapy was initiated. Early physiotherapy focused on cryotherapy and isometric exercises for the quadriceps, all while the patient remained non-weight bearing and the knee immobilised in a backslap cast for 14 days.

At the first follow-up visit, the initial cast was replaced with a hinged knee brace set to allow 0 to 30 degrees of flexion for the next two weeks. Every two weeks thereafter, the range of flexion was increased by an additional 30 degrees. Full weight bearing was not permitted until radiographs confirmed sufficient fracture healing, usually around six weeks after surgery.

Once the patient no longer required the support of the brace, they began full range-of-motion exercises, as well as progressive strengthening of the quadriceps and hamstrings using isometric and active-assisted techniques. By the eighth week postoperatively, the rehabilitation program expanded to include closed-chain strengthening exercises, such as cycling and leg presses, supporting a gradual return to full function.

## Discussion

Literature review and current practice

Multiple surgical techniques have been described for the management of tibial eminence fractures in pediatric patients [1], with the primary goal of achieving stable anatomic reduction while minimising the risk of growth plate injury. Traditional methods, such as cannulated screw fixation, have demonstrated excellent mechanical stability but carry a risk of physeal violation (Table 1). Similarly, arthroscopic suture fixation techniques that involve transphyseal tunnels also present a potential for growth disturbances (Table 1) [2]. 

**Table 1:** Comparison of current techniques and our method. ACL: anterior cruciate ligament; PEEK: polyether ether ketone

Feature	Traditional Screw Fixation [‎1,‎4,‎7]	Arthroscopic Suture Fixation (Transphyseal) [‎2,‎5‎,8]	Arthroscopic Suture Bridge (Skyway, etc.) [‎9]	Our Novel Physeal-Sparing Technique
Fixation method	Cannulated screw(s) through the fracture fragment	Sutures passed through the ACL and tibial tunnel(s) across the physis	Knotless anchors + suture bridge across fragment	High-tensile sutures through ACL + subcapsular routing to distal tibia anchors
Growth plate involvement	Yes (risk of physeal injury)	Yes (drilling through physis)	No (anchors distal to physis)	No (anchors distal to physis, no tunnel through growth plate)
Anatomical footprint restoration	Good if well-reduced	Variable, depends on tunnel location	Good with a wide suture bridge	Excellent, with posterior suture positioning optimising footprint restoration
Reduction assistance	Open or arthroscopically assisted	Arthroscopic (direct manipulation)	Arthroscopic with suture tensioning	Arthroscopic; direct reduction + suture traction and posterior push manoeuvre
Risk of hardware removal	Yes (hardware prominence, growth issues)	No	No	No
Technical difficulty	Moderate (open/arthroscopic)	Moderate (requires drilling skills)	High (precise anchor placement)	Moderate to high (requires subcapsular tunnel creation, suture management)
Risk of fragment comminution	Higher (screw insertion in fragile fragment)	Moderate	Low	Low
Soft tissue management	Less focus	Moderate; sutures pass through the ACL	High; sutures bridge the ACL and the fragment	High; sutures pass posteriorly around ACL, minimising ACL trauma
Use of fluoroscopy	Recommended for screw placement	Required for tunnel placement	Limited (anchor verification)	Essential to verify anchor positioning distal to the physis
Implant used	Screws (metallic or bioabsorbable)	High-strength sutures + tibial button or knot	All-suture anchors (knotless)	PEEK SwiveLock anchors (knotless)

In response to these challenges, physeal-sparing approaches have been increasingly adopted. Techniques such as the arthroscopic suture bridge method (e.g., the Skyway technique) and all-suture knotless anchor methods have been developed to preserve the physis while providing stable fixation (Table 1). These techniques emphasise minimally invasive principles and avoid hardware crossing the growth plate [3,4].

Our novel technique aligns with this modern trend by utilising arthroscopically placed high-tensile sutures routed subcapsularly and fixed to the anterior tibial surface distal to the physis using knotless polyether ether ketone (PEEK) anchors (Arthrex). Key distinguishing features of our approach include posterior suture placement around the ACL to improve the vector of reduction and the strategic use of a subcapsular tract to avoid drilling through the fracture bed [5].​ A comparison of current techniques and our method is outlined in Table 1 [6,7].

Obstacles and pitfalls

Achieving accurate reduction of the avulsed tibial eminence fragment remains one of the primary challenges associated with this technique (Table 2). Several factors contribute to this difficulty, including the inherent characteristics of the fracture itself, such as extensive comminution, fracture bed irregularity, loose fragments, and interposition of soft tissues like the intermeniscal ligament or parts of the lateral meniscus [4]. To address these challenges, meticulous preparation of the fracture bed is critical. Thorough debridement using a bone curette and an arthroscopic shaver, along with removing any interposed soft tissues, should be performed before attempting reduction.

**Table 2:** Common technical pitfalls. ACL: anterior cruciate ligament; PEEK: polyether ether ketone Adapted from references [6,9].

Category	Specific Issue	Cause	Effect	Solution
Fracture reduction	Difficult fragment reduction	Extensive comminution, fracture bed irregularity, loose fragments, interposed intermeniscal ligament or meniscal tissue	Difficulty in achieving anatomical reduction	Thorough fracture bed preparation with arthroscopic shaver and bone curettes; removal of interposed tissues
Force vector	Unfavourable vector of pull (anterior and distal traction)	Avoidance of transphyseal drilling alters the traction vector	Posterior lift-off of the fragment, especially if the posterior part is unstable	Position sutures as posteriorly as possible around the ACL; use a blunt instrument (trocar/Frazier) to push the fragment posteriorly before tensioning sutures
Anchor placement	Anchor placed too close to the growth plate	Incorrect anchor trajectory or insufficient clearance from physis	Risk of physeal injury	Routine intraoperative fluoroscopy to confirm anchor clearance; careful insertion of 4.75 mm × 19.1 mm PEEK SwiveLock anchor distal to physis

Another intrinsic limitation of this technique stems from the deliberate avoidance of transphyseal drilling. While this strategy effectively preserves the proximal tibial physis, it alters the direction of traction forces. Specifically, suture tension may generate an anterior and distal pull vector, potentially leading to posterior fragment lift-off, particularly if the posterior aspect of the fragment is unstable [8]. To mitigate this risk, sutures should be positioned as posteriorly as possible around the ACL to optimise the direction of pull. Additionally, intraoperative use of a wide blunt instrument, such as a trocar or a Frazier elevator, can assist in pushing the fragment posteriorly into its anatomic bed before tensioning the sutures under the capsule (Table 2). Despite these mechanical challenges, with careful surgical technique and experience, we have consistently achieved satisfactory anatomical reduction and restoration of the ACL footprint.

A common technical pitfall involves the incorrect placement of the knotless anchor too close to the growth plate, particularly if the anchor is inadvertently directed toward the physis. In our practice, we utilise a 4.75 mm × 19.1 mm PEEK SwiveLock anchor for suture fixation. To avoid physeal injury, ensuring adequate clearance and a proper insertion trajectory is critical. We recommend routine use of intraoperative fluoroscopic guidance to confirm safe and accurate anchor placement (Table 2).

Limitations

This study has several limitations. The sample size is relatively small, which may limit the generalizability of the findings. Additionally, the absence of a control group prevents direct comparison with other established techniques. The follow-up duration, although sufficient to assess early functional outcomes and return to sport, may not be adequate to fully evaluate long-term complications such as growth disturbances or post-traumatic osteoarthritis. Finally, this technique was performed by experienced arthroscopic surgeons at a single centre, which may limit reproducibility in other settings.

## Conclusions

Our novel physeal-sparing fixation technique offers a reproducible, minimally invasive method for treating tibial eminence fractures in skeletally immature patients. By avoiding transphyseal drilling and employing posterior suture placement with subcapsular routing, the method preserves growth plate integrity while ensuring stable anatomical reduction. Early clinical outcomes demonstrate excellent fracture healing, restoration of knee stability, and safe return to sport. Further studies with larger cohorts and longer follow-up are warranted to validate these promising results.
